# Generative lesion pattern decomposition of cognitive impairment after stroke

**DOI:** 10.1093/braincomms/fcab110

**Published:** 2021-05-22

**Authors:** Anna K Bonkhoff, Jae-Sung Lim, Hee-Joon Bae, Nick A Weaver, Hugo J Kuijf, J Matthijs Biesbroek, Natalia S Rost, Danilo Bzdok

**Affiliations:** 1 Department of Neurology, J. Philip Kistler Stroke Research Center, Massachusetts General Hospital, Harvard Medical School, MA, Boston, USA; 2 Department of Neurology, Hallym University Sacred Heart Hospital, Hallym Neurological Institute, Hallym University College of Medicine, Anyang, Republic of Korea; 3 Department of Neurology, Cerebrovascular Center, Seoul National University Bundang Hospital, Seoul National University College of Medicine, Seongnam, Republic of Korea; 4 Department of Neurology and Neurosurgery, UMC Utrecht Brain Center, University Medical Center Utrecht, Utrecht, the Netherlands; 5 Image Sciences Institute, University Medical Center Utrecht, Utrecht, the Netherlands; 6 Department of Biomedical Engineering, McConnell Brain Imaging Centre, Montreal Neurological Institute, Faculty of Medicine, School of Computer Science, McGill University, Montreal, Canada; 7 Mila—Quebec Artificial Intelligence Institute, Montreal, Canada

**Keywords:** ischaemic stroke, hemisphere-aware analysis, clinical outcome prediction, Bayesian hierarchical modelling, machine learning

## Abstract

Cognitive impairment is a frequent and disabling sequela of stroke. There is however incomplete understanding of how lesion topographies in the left and right cerebral hemisphere brain interact to cause distinct cognitive deficits. We integrated machine learning and Bayesian hierarchical modelling to enable a hemisphere-aware analysis of 1080 acute ischaemic stroke patients with deep profiling ∼3 months after stroke. We show the relevance of the left hemisphere in the prediction of language and memory assessments and relevance of the right hemisphere in the prediction of visuospatial functioning. Global cognitive impairments were equally well predicted by lesion topographies from both sides. Damage to the hippocampal and occipital regions on the left was particularly informative about lost naming and memory functions, while damage to these regions on the right was linked to lost visuospatial functioning. Global cognitive impairment was predominantly linked to lesioned tissue in the supramarginal and angular gyrus, the post-central gyrus as well as the lateral occipital and opercular cortices of the left hemisphere. Hence, our analysis strategy uncovered that lesion patterns with unique hemispheric distributions are characteristic of how cognitive capacity is lost due to ischaemic brain tissue damage.

## Teaser

Bonkhoff et al. integrate machine learning and Bayesian hierarchical modelling to enable hemisphere-aware analysis of 1080 stroke patients. They quantify lateralization effects of cortical and subcortical regions, such as the pallidum and hippocampus, of middle and posterior cerebral artery vascular territories to the left for naming/memory and to the right for visuospatial functions.

## Graphical Abstract

**Graphical Abstract:**
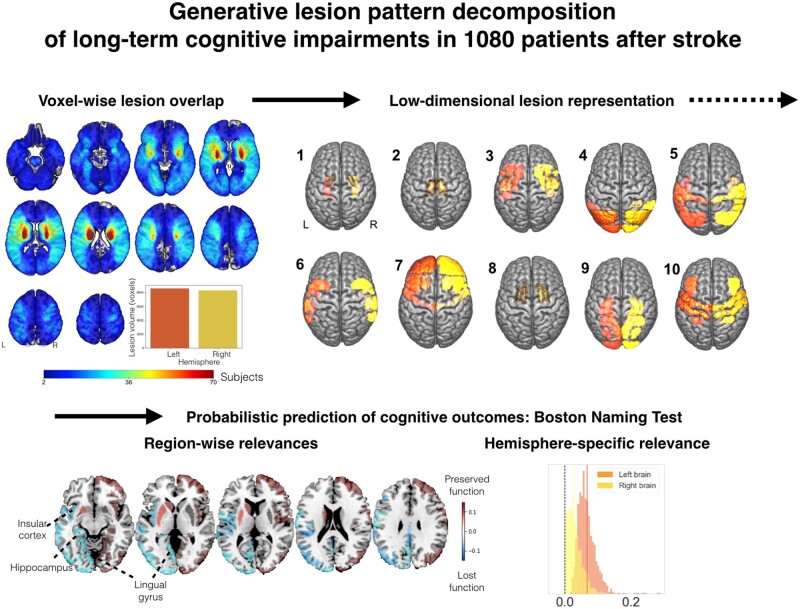

## Introduction

In the USA, somebody experiences a stroke every 40 s.^1^ Ensuing ischaemic brain lesions do not only underly motor impairments but also cause a continuum of cognitive impairments ranging from clinically silent to overtly disabling symptoms.^2^ Variability in cognitive consequences underscore the clinical importance of an early and accurate prediction of cognitive outcomes at an individual patient level. In particular, an augmented understanding of the precise structure–function correspondence of how the spatial distribution of stroke lesions are linked to distinct cognitive deficits will pave the way for such reliable predictions of clinical endpoints.

Many brain functions involve various lower and higher cognitive processes and are supported by regions spread across the brain’s grey matter. A key aspect of the spatial distribution of neurocognitive processes is the preferential lateralization to one hemisphere^3^. Two of the potentially most consistent aspects of hemispheric functional lateralization, language^4^^,^^5^ and attention,^6^ may distinctly relate to the left versus right hemisphere^7^. Indeed, language impairments occur more frequently and severely after left- and neglect after right-hemispheric strokes.

Previous stroke lesion studies have oftentimes been conducted under some anatomical constraint or deliberate topographical focus to tame the amount of information in lesion volume and distribution.^8^ Hence, it has become a widely accepted practice that language impairments and neurological neglect are exclusively studied in the expected predominantly affected hemisphere (i.e., language impairment in the left hemisphere,^9^^,^^10^ neglect in the right hemisphere^11^^,^^12^). For instance, one of the earliest studies on neglect examined overlay lesion maps of 110 patients, all with only right-sided stroke.^11^ Nonetheless, several studies suggest that acute neglect also affects more than 20% of left-hemispheric stroke patients.^13^ In a similar vein, aphasia is mostly caused by lesions in the hemisphere contralateral to an individual’s dominant hand. However, there are cases of ‘crossed aphasia’,^14^ implying language deficits due to lesions in the right hemisphere, *ipsilateral* to the dominant hand. As such, many existing brain-imaging studies on stroke have studied only a single brain hemisphere.

Examples of lesion-symptom mapping studies, that have embraced the full complexity of lesion topographies and investigated left- and right-hemispheric stroke concurrently, exist.^15–17^ Nonetheless, the applied techniques did not typically offer a principled means to quantify and carefully compare hemisphere-specific contributions to clinical outcome prediction.

Therefore, in this proof-of-principle study, we sought to integrate machine learning and Bayesian hierarchical modelling to enable a hemisphere-aware analysis of 1080 ischaemic stroke patients. By bringing to bear this novel statistical framework, we investigated lesion associations to cognitive outcomes obtained on average 3 months after stroke and sought to disentangle lateralization effects on the level of entire hemispheres, specific lesion patterns and individual brain regions. We concentrated on four key cognitive outcomes that captured global cognitive abilities, as well as more language- and visuospatial-attention-related functions. We hypothesized to find a left-hemispheric dominance for language-centred tests, and right-hemispheric dominance for tests more heavily relying on attention and visuospatial abilities.

## Materials and methods

### Participant recruitment

Using a prospective stroke registry database,^18^ we identified hospitalized stroke patients who were diagnosed with acute ischaemic infarction based on diffusion-weighted MRI typically within 1 week after symptom onset between January 2007 and December 2018 in Hallym University Sacred Heart Hospital or Seoul National University Bundang Hospital, South Korea. Among them, a total of 1080 patients were selected based on the following criteria: (i) availability of brain MRI showing the acute symptomatic infarct(s) on diffusion-weighted imaging (DWI) and/or fluid-attenuated inversion recovery, (ii) successful infarct segmentation and registration (see below), (iii) no previous cortical infarcts, large subcortical infarcts (>15 mm) or haemorrhages (>10 mm) on MRI and (iv) availability of data on key demographics and neuropsychological assessment (the 60-min Korean-Vascular Cognitive Impairment Harmonization Standards-Neuropsychology Protocol, K-VCIHS-NP)^19^^,^^20^ within 1 year post-stroke. We excluded patients (i) whose MRI was inadequate for properly obtaining neuroimaging variables, (ii) who had bilateral stroke and (iii) inability to undergo cognitive testing due to severe aphasia, as determined by the attending physician.

Stroke subjects provided informed written consent in accordance with the Declaration of Helsinki. The local institutional review boards approved the study protocol and gave waived consent requirements based on the retrospective nature of this study and minimal risk to participants.

### Characteristics of participant sample

Stroke patients underwent a comprehensive battery of neuropsychological tests ∼3 months after the onset of acute stroke (median time post-stroke: 98 days).^20^ We here predominantly focused on four key assessments of post-stroke cognitive performance: Korean Mini-Mental State Examination [total score, (MMSE)],^21^ language performance [total score, Korean short version of the Boston Naming Test (BN)],^22^ memory function [Immediate recall, Seoul Verbal Learning Test (SVL), the Korean equivalent to the California Verbal Learning Test]^20^ and visuospatial functioning [Rey Complex Figure Test (RC)].^23^ Performance in the MMSE reflects global cognition including the orientation to time and place, as well as calculation or language performance. The BN (Korean short version) is a clinical standardized test that measures the naming abilities of patients. The SVL, in turn, examines episodic memory performance and requires the auditory learning of a word-list and tests its memorization by an immediate recall task. In the RC, patients are asked to first copy a complex line drawing. In this copying phase, the test requires the recognition of patterns and details and thus captures visuospatial abilities.

The cognitive performance prior to stroke onset was captured by the Informant Questionnaire on Cognitive Decline in the Elderly.^24^ This test provides a widely used and validated measurement instrument of cognitive decline in the 10 years prior to stroke onset and relies on health-proxy reports. Additionally, available sociodemographic and clinical information included: age, sex, time since stroke onset (in days), years of education and lesion volume (grey and white matter). Continuous (non-binary) variables were put on a more comparable scale by normalization (MMSE, BN, SVL, RC, age, time since stroke onset, lesion volume, education years and Informant Questionnaire on Cognitive Decline in the Elderly). The covariate indicating the exact time after stroke onset was included to address the time-dependent likelihood of cognitive impairments after stroke.^25^ In sensitivity analyses, we restricted the sample to only those patients 65 years and older and furthermore simultaneously analysed performances in the BN and SVL tests, given their expected high correlation.

### Neuroimaging data pre-processing

Brain-imaging acquisition was typically performed within the first week after stroke. Brain scanning included structural axial T1, T2-weighted spin echo, fluid-attenuated inversion recovery and DWI sequences (3.0 T, Achieva scanner, Philips Healthcare, Netherlands, image dimensions: 182 × 218 × 182; c.f., Supplementary material for details). Stroke lesions were manually segmented on DWI or less frequently fluid-attenuated inversion recovery images by experienced, trained investigators (A.K.K. and G.A.) relying on in-house developed software based on MeVisLab (MeVis Medical Solutions AG, Bremen, Germany).^26^ Lesion segmentations were successively checked and adapted by two experienced raters (N.A.W. and J.M.B). Subsequently, images as well as corresponding lesion maps were linearly and non-linearly normalized to Montreal Neurological Institute (MNI-152) space employing the RegLSM image processing pipeline (public code: http://lsm.isi.uu.nl/).^27^ Quality of normalization was rigorously controlled by an experienced rater (N.A.W.). If there were any visual differences between the original and registered lesion maps during quality control, normalized lesion maps were manually corrected.

To develop a fully probabilistic generative modelling framework specifically tailored to stroke populations, we combined: (i) unsupervised dimensionality reduction of lesion maps and (ii) Bayesian hierarchical modelling to predict the outcome in cognitive performance tests. We here focused on quantitative analyses of the 3-months outcomes measured on the MMSE, Korean short versions of the BN and the SVL, as well as the RC.

### Data-driven exploration of anatomic lesion representations

Each to-be-analysed lesion map featured 435 642 total voxels of 1 mm^3^ in grey matter. We sought to explore coherent topographical patterns that may be hidden in these high-dimensional brain scans and to represent each patient’s lesion fingerprint in a simpler and more directly interpretable form. To this end, we initially computed the lesion load within 54 parcels (108 for both hemispheres), composed of the Harvard-Oxford cortical atlas with 47 regions and subcortical atlas with 7 regions in each hemisphere.^28^ We first counted the number of voxels affected per atlas-defined brain region. In doing so, we obtained 54 regional measures of lesion load per hemisphere in each participant. We log-transformed and concatenated the ensuing lesion load measures for the left and right hemisphere. We then applied non-negative matrix factorization (NMF)^29^ as a multivariate encoding strategy to identify 10 unique combinations of spatially distributed region damage that we will call *lesion atoms* in the following. The number of 10 lesion atoms per hemisphere was chosen to achieve a balance between capturing a substantial amount of lesion variability, on the one hand, and keeping the number of quantities low for neuroscientific domain interpretation, on the other hand.

NMF achieved a low-rank approximation of the anatomical lesion data by partitioning the lesion maps into a matrix of latent factor representations **W**. These hidden topographical representations link the emerging spatial lesion pattern topography to each of the original anatomical brain regions. The matrix of latent factor loadings **H** indicates how relevant each emerging spatial lesion pattern is to describe a specific patient’s overall lesion distribution. The atomic representations hence decompose the actual lesion constellation in a given patient given by **V** = **WH**, with **V** being the local lesion summaries of p × n dimensions (p = number of brain regions and n = number of subjects). Accordingly, **W** and **H** have p × k and k × n dimensions, respectively, with k representing the number of latent spatial patterns.

NMF provided at least two key advantages in our modelling scenario: In contrast to classical lesion-symptom mapping,^9^ that considers the effect of one location at a time, each brain location could here belong to several latent lesion components of **W** to varying degrees. In this way, each location could contribute to the prediction of cognitive outcome through relative contributions of multiple components, each of which reflected extracted lesion archetypes distributed across the whole brain. In this way, it was more similar to multivariate-lesion symptom mapping approaches^12^^,^^15^^,^^16^ and also alleviates the likelihood and amount of distorted functional localization due to functional dependence.^30^^,^^31^ Additionally, the non-negativity of the segmented brain lesion information and the non-negativity constraint of the NMF model ensured the automated derivation of a parts-based representation. That is, each individual latent component **W_a_** represented a unique and directly interpretable aspect of the overall topographical lesion pattern variation. The neurobiologically interpretable sum-of-parts representation enabled by NMF is in contrast to latent representations learned by alternative matrix factorization algorithms. For instance, in principal component analysis, individual lesions would be recovered through convoluted additions and subtractions of several components with positive and negative weights. For this reason, the overall effect of all principal components, yet not the effect of ensuing *individual* components, would have been as easily and intuitively interpretable to draw neuroscientific conclusions.

### Predicting interindividual differences in cognitive outcomes

The NMF-derived expressions of topographical lesion atoms provided the neurobiological input into our Bayesian hierarchical model^32^ to explain interindividual differences in cognitive outcome scores. We opted for this generative, multi-level approach given our primary motivation to quantify the full probabilistic information in our model parameter estimates at several neurobiological organizational levels. In particular, the obtained uncertainty distributions therefore made explicit the confidence of the model for each hemisphere and each lesion atom in contributing to the successful prediction of a given cognitive outcome. We have designed a bespoke Bayesian hierarchical model dedicated for each cognitive outcome (i.e., MMSE, BN, SVL or RC).

To directly examine possible differences in hemispheric predictive relevance in our data, the modelled generative process assumed a joint dispersion prior for all lesion atoms of each hemisphere. Therefore, the standard deviation priors for the left and right hemispheres could capture the hemisphere-specific predictive contributions tiled across all candidate lesion atoms. Priors of left- and right-hemispheric standard deviations were additionally combined through a joint hyperprior to complement the hierarchical model structure. Furthermore, the model took into account several covariates, including age, age^2^, sex, age–sex interactions, time since stroke onset, education years, pre-morbid cognitive performance and total lesion volume (in case of MMSE, BN and SVL). The RC model was unstable upon inclusion of the lesion volume covariate; thus we here present model results that only take into account region-wise lesion damage, yet not total lesion volume. We could then inspect these covariates in conjunction with hemisphere or lesion atom contributions to concurrently capture the contribution of a lesion representation to the cognitive outcome while rendering explicit the influence of important demographic, sociodemographic and clinical factors (c.f., Supplementary Fig. 1 for the full model specification).

Samples from the joint posterior distribution of the model parameters were drawn by the No U-Turn Sampler, a kind of Monte Carlo Markov Chain algorithm (setting: draws = 3500).^33^ Posterior predictive checks were carried out after model estimation to evaluate the obtained predictive model (with respect to its *R*^2^-based explained variance). That is, we empirically assessed the simulated outcome predictions generated by our model solution to approximate external validation based on our patient sample.

Our Bayesian hierarchical approach facilitated the careful dissection of predictive relevance allocated to different levels of the model. For all outcome models, we first evaluated lateralization effects that were inferred from the left and right hemisphere posterior dispersion distributions. In analogy to ANOVA applications, where the total variance of a specific outcome variable is partitioned into several sources of variance, we thus here focused on the proportion of the variance that was attributable to variation in all lesion atoms linked to one brain hemisphere. In this way, we capitalized on the general hemispheric importance in predicting the outcome, yet de-emphasized the directionality of these associations: ‘Did regional effects from either one hemisphere, the left or right one, contribute more substantially to the accurate prediction of the outcome of interest?’ Therefore, predictive relevance could originate from predictors of more (‘more impaired’) or less deteriorated (‘less impaired’) cognitive impairment observed in our patient sample. Subsequently, we considered lateralization effects of specific lesion atoms and lastly reverted back the predictive relevance of lesion atoms^34^ to the level of the anatomical brain regions for each of the cognitive outcomes.

### Code availability

Analyses were conducted in a Python 3.7 environment and predominantly relied on the packages nilearn and pymc3. Full code is available here: https://github.com/banilo/BrainCommunications2021/

### Data availability

Anonymized data that support the findings of this study are available from the corresponding author upon reasonable request.

## Results

### Characteristics of cognitive outcomes and patient sample

We relied on structural MRI data of 1080 ischaemic stroke patients, typically obtained within one week after symptom onset, to predict cognitive outcomes ∼3 months post-stroke. Analyses were focused on the Korean MMSE (total score, 1076 patients),^21^ language performance (total score, Korean short version of the BN, 1061 patients),^22^ memory function (immediate recall, SVL, 1065 patients)^20^ and visuospatial functioning (Copying, RC, 1002 patients).^35^ Patients scored 24 points on the MMSE on average. Out of 15 objects, patients correctly named on average 10 objects in the BN. In the SVL, patients could remember 16 out of 36 words on average and 26 points were recorded in the RC on average. Moreover, patients had an average of 9.3 ± 0.3 years of education and scored 3.38 ± 0.04 points on the Informant Questionnaire on Cognitive Decline in the Elderly (Table 1).^24^

**Table 1:** Patient characteristics

	Mean (95% CI)
Age	67.4 ± 0.7 (range 31–94; median 69.0)
Sex	57% male, 43% female
Clinical history of stroke	12.6 %
Years of education	9.3 ± 0.3 (range 0–23; median 9)
IQCODE	3.38 ± 0.04 (range 1.3–5.0; median 3.2)
Mini-mental state examination (total score)	24.0 ± 0.4 (range 0–30; median 26)
Boston naming test (total score)	10.1 ± 0.2 (range 0–15; median 11)
Seoul verbal learning test (immediate recall)	15.2 ± 0.4 (range 0–36; median 16)
Rey complex figure test (copy)	26.3 ± 0.55 (range 0–36; median 30)

### Anatomy of the extracted lesion atoms in stroke patient

**Figure 1:**
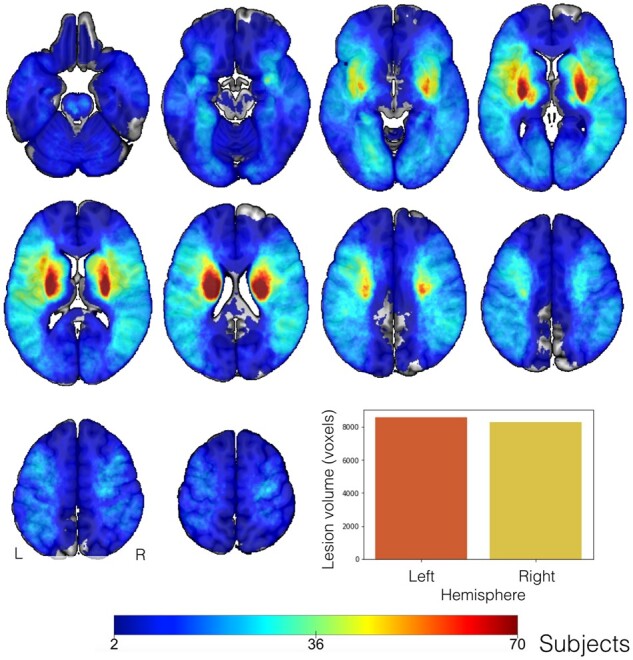
**Topographical overlap of ischaemic stroke lesions in 1401 patients in Montreal Neurological Institute reference space before the exclusion of patients with bilateral stroke.** Tissue damage caused by neurovascular ischaemic events affected predominantly subcortical regions, in line with previous reports.^36^ The strongest anatomical overlap in tissue lesions was found in the vascular territory supplied by the left and right middle cerebral arteria. We confirmed excellent whole-brain coverage, with pronounced inter-individual heterogeneity in topographical distribution of tissue lesions. The overall lesion volume did not differ significantly between the left and right hemisphere in our patient cohort (*t*-test: *P *=* *0.81, bar plot lower right corner).

Voxel-wise topographical overlap of the lesion distributions demonstrated an excellent whole-brain coverage across patients (Fig. 1). In the majority of the patients, stroke affected either the left or right vascular territory of the middle cerebral artery. The maximum of lesioned tissue was localized in subcortical zones. Importantly, there was no difference in the number of lesioned voxels per patient between the left versus right hemisphere (two-sided *t*-test: *P *=* *0.81).

**Figure 2:**
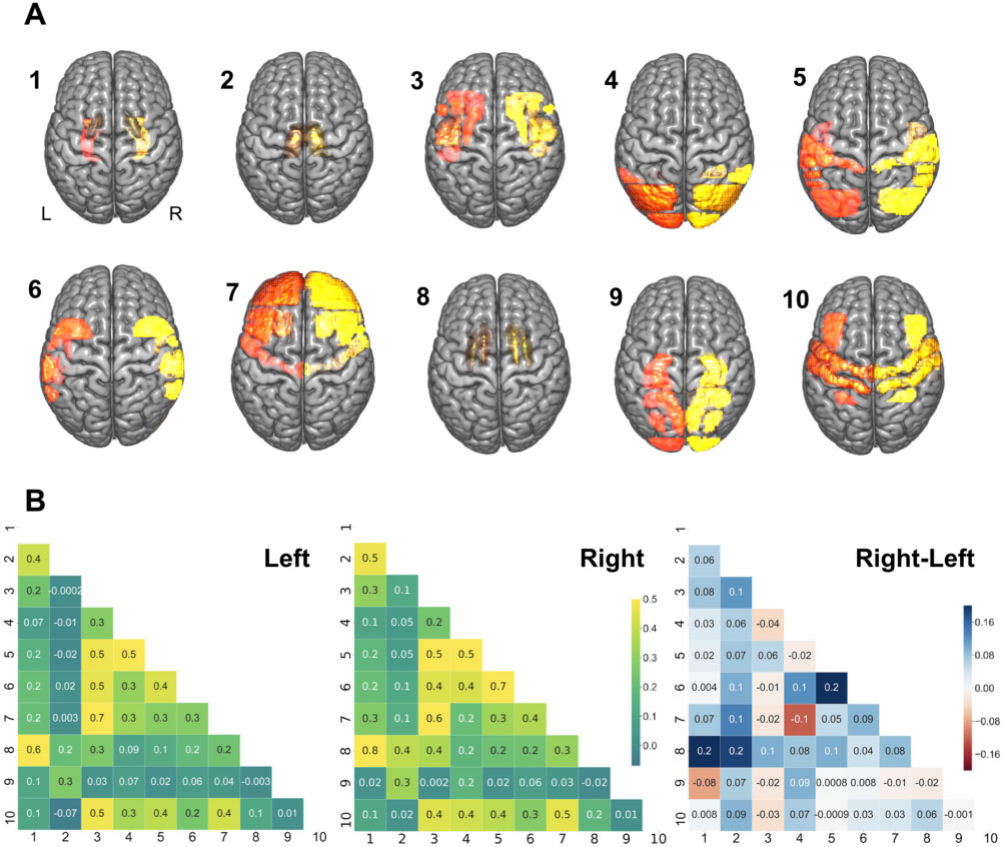
**Lesion atoms of stroke patterns were derived by dimensionality-reducing pattern recognition.** Voxel-wise lesions were summarized based on 108 brain region definitions (54 per hemisphere). The region-wise lesion measures were then further compressed into 10 essential lesion-pattern dimensions on each side by capitalizing on non-negative matrix factorization. (**A**) The thus uncovered set of lesion atoms recapitulated several well-documented stroke lesion patterns and was further supported by biological plausibility of the lesion pattern topography. For instance, several lesion atoms delineated specific cortical and subcortical stroke patterns coherent with the anatomy of the middle cerebral artery (subcortical: lesion atom 1, 2, 8; cortical: 3, 5, 6, 10). Posterior cerebral artery strokes were appropriately captured by lesion atom 9. Lesion atom 7 depicted most topographies in line with strokes of the anterior as well as middle cerebral artery. In particular, lesion atom 7 (anterior cerebral artery and middle cerebral artery) delineated the frontal pole, middle and inferior frontal gyrus, precentral gyrus, frontal orbital cortex and frontal opercular cortex. Lesion atom 9 (posterior cerebral artery) included regions of the inferior temporal gyrus, intracalcarine cortex, cingulate gyrus, precuneus and cuneal cortex, parahippocampal gyrus, lingual gyrus, temporal and occipital fusiform cortex, occipital pole, as well as hippocampus. The remaining lesion atoms (largely middle cerebral artery) captured lesion variation in regions including the subcortical regions globus pallidus, putamen, caudate and thalamus, as well as insular and cortical middle cerebral artery regions (middle and inferior frontal gyrus (pars opercularis), superior, middle and inferior temporal cortex, pre- and post-central gyrus, opercular cortex (frontal, central, parietal), planum polare, Heschl’s gyrus, angular gyrus, supramarginal gyrus, superior parietal lobule, lateral occipital cortex and the occipital pole). Additionally, the topography of lesion atoms aligned with a certain vessel territory matched the frequency of its affection by ischaemic stroke in clinical practice: we obtained more fine-grained subdivisions of stroke topographies due to middle cerebral artery strokes in view of the high frequency that this vascular supply territory was affected. *Left/right* hemispheric lesion atoms shown in orange/yellow. (**B**) Lesion atoms exhibited only small hemispheric differences in pattern correlations. Lesion atoms in subcortical areas were particularly often simultaneously affected in patients and thus strongly mutually related in the left and right hemisphere (atoms 1, 2, 8). For lesions on the right side, putamen and caudate (atom 8) exhibited slightly more prominent correlations with pallidal (atom 1) and thalamic areas (*darker blue colour*).

We summarized the initial high-dimensional lesion information at the voxel level in 108 cortical and subcortical brain regions for the exploration of coherent hidden patterns of lesion topography. We then uncovered a set of distinct topographical lesion configurations, *lesion atoms*, in a data-driven fashion based on NMF.^29^ The spatial distribution of the lesion atoms corresponded to interpretable and biologically plausible components of stroke lesions and correlation patterns between lesions atoms were largely similar between the left and right hemispheres (Fig. 2). Lesion atoms separately represented topographies in line with territories of arterial blood supply via the anterior (lesion atom 7), middle (cortical: 3, 5, 6, 10, subcortical: 1, 2, 8) and posterior (PCA, 9) cerebral artery territories. The Pearson correlation between the original and after NMF-dimensionality reduction reconstructed 108-dimensional region-wise lesion representations amounted to *r *=* *0.9.

After extracting this set of coherent atomic patterns that together underlie stroke topographies, we investigated the differential effects of (i) hemispheres, (ii) lesion atoms and (iii) anatomical regions on the cognitive outcome after stroke.

### Hemisphere relevance for clinical outcome

**Figure 3:**
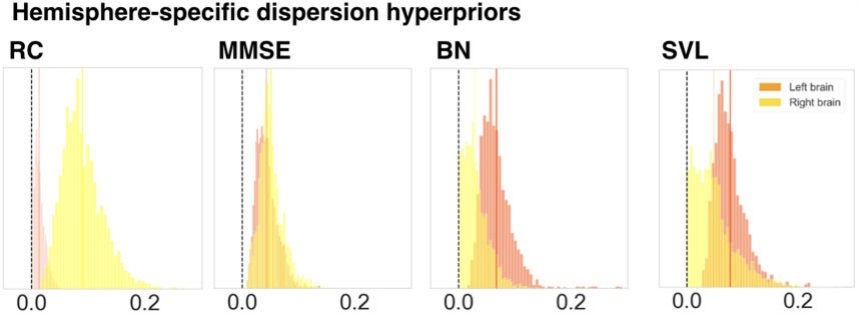
**Hemispheric lateralization effects for predicting clinical outcomes in stroke patients.** Displays the relevance of the posterior parameter distributions of the left and right brain. These were obtained through four Bayesian hierarchical models dedicated to predicting four cognitive outcomes. In case of the Mini-Mental State Examination (MMSE), often considered a measure of global performance tapping into various cognitive domains, lesions in the left and right brain contributed equally to prediction success. In contrast, lesions in the left brain were more relevant for single-patient predictions of cognitive outcomes in case of the Boston Naming (BN) and Seoul Verbal Learning Test (SVL) and lesions in the right brain were more relevant for performance in the Rey Complex Figure Test (RC). The shown posterior model parameters correspond to the upper hemisphere level of our Bayesian multi-level modelling strategy that uncovered the hemisphere-specific model certainty for each cognitive performance dimension. The revealed lateralization effects for BN, SVL and RC highlight the left and right hemisphere (c.f., *vertical, darkorange/yellow lines for means of left/right-hemispheric means of the posterior distributions*) that suggest a dominance of left- or right-hemispheric lesion information in driving predictions for memory, verbal learning and visuospatial functioning outcomes.

We first examined possible hemisphere-specific effects on cognitive impairment in our stroke patients. We specifically investigated whether lateralized relevance varied across the four outcome scores. We relied on the posterior distribution of variance parameters at the hemisphere level that had inferred the magnitude of hemisphere-specific relevance for a given cognitive dimension. In the case of MMSE, similarly distributed hemisphere posteriors suggested that the combination of all lesion atoms of one hemisphere were equally relevant for predicting global cognitive outcome (mean of the left hemisphere-level posterior distribution = 0.0432, highest probability density interval of the left hemisphere-level posterior distribution covering 94%-certainty (HPDI) = 0.0117–0.078; right posterior mean = 0.0513, HPDI = 0.0126–0.0901, Fig. 3, middle left plot). While the left hemisphere was predictive of poorer MMSE performance, the right hemisphere was predictive of more preserved MMSE performance. In contrast, the left hemisphere showed a markedly higher predictive relevance for our analysis of BN and SVL (BN: left posterior mean = 0.0673, HPDI = 0.0217–0.113, right posterior mean = 0.0299, HPDI = 0.0005–0.0682; SVL: left posterior mean = 0.0776, HPDI = 0.0352–0.132, right posterior mean = 0.0485, HPDI = 0.0004–0.117, Fig. 3, middle right and right plot). That is, tissue lesions located in the left hemisphere contributed comparatively more to the predictability of the naming and verbal learning impairments. The opposite was true for RC: Here, the right hemisphere demonstrated a distinctly higher predictive relevance (left posterior mean = 0.0132, HPDI = 0.0–0.0301, right posterior mean = 0.0898, HPDI = 0.0281–0.156, Fig. 3, left plot). For BN, SVL and RC, either the left or the right posterior distributions of the hemisphere variance parameter had a higher posterior mean as well as wider spread across all lesion atoms. Hence, the hemisphere parameters in our multi-level Bayesian model were directly informative about the left- or right-hemispheric dominance when explaining interindividual variation in cognitive performance profiles.

**Figure 4:**
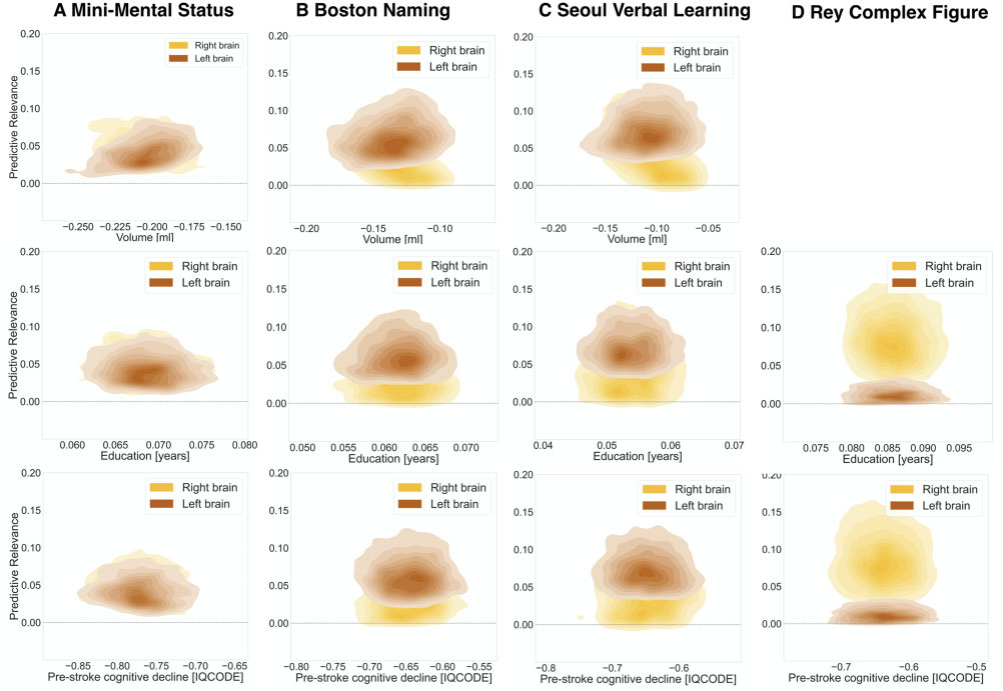
**Hemispheric relevance depends on key covariates.** Inter-relation between the relevance of lesion load in a given hemisphere (*y-*axis*, left hemisphere: moccasin, right hemisphere: yellow*) and marginal posterior parameters of key covariates (*x*-axis) in predicting cognitive performance. Lesion volume, as well as all four outcome variables were normalized to the same z-score scale. Despite assumed important interactions,^37^ neither one of typically applied uni- or multivariate models represents an integrative approach to jointly study lesion location and further sociodemographic and clinical covariates. We here present such a joint analysis that is possible based on the combination of our large sample size and generative modelling framework. *Top row:* The influence of lesion volume on the cognitive outcome prediction varied across the three outcome scores: A one standard unit increase in lesion volume was sufficient to cause a 21% standard unit decrease in MMSE performance. Conversely, one standard unit increases in lesion volume led to only 11–14% standard unit decreases in BN and SVL performances. RC is not shown, as lesion volume was not included as covariate in RC models. *Middle row:* Years of education had positive effects of comparable magnitude on MMSE, BN and SVL outcome scores: An additional year of education predicted a 5–7% standard unit increase. This effect was even more pronounced for RC, where an additional year of education predicted an increase of 8.5%. *Bottom row:* One point more on the IQCODE scale, i.e. a higher pre-stroke cognitive decline, predicted a ∼65% drop in BN, SVL and RC standard unit performance. In case of MMSE, on more IQCODE point resulted in an even higher decrease of 77% standard unit performance.

Furthermore, we observed characteristic inter-relations between these hemisphere-specific effects and important covariates, i.e., lesion volume, education attainment and pre-stroke cognitive performance, in the prediction of cognitive outcome (Fig. 4). We found that lesion volume and a more deteriorated pre-stroke cognitive performance led to decreases in performance on any of the four tests. In contrast, more years of education supported a more preserved performance. Effects of total lesion volume and pre-stroke cognitive performance were the most pronounced for MMSE performance and years of education had the highest relevance for RC performance. These effects were coupled with hemisphere effects that were balanced (MMSE) or more pronounced on the left (BN, SVL) or right (RC).

### Lesion atom relevance for clinical outcome

The level of lesion atoms in our hierarchical Bayesian model offered yet another opportunity to gain rich insights on lateralization effects linked to not only *entire* hemispheres, but specific lesion topographies *within* a hemisphere in the prediction of cognitive outcome. We thus direct our attention to the examination of individual lesion atoms that combined several brain regions. We inferred lateralization effects from non-overlapping posterior parameter distributions of left and right lesion atoms. Such non-overlapping distributions implied that a specific lesion atom in either the left or right hemisphere was reliably more predictive of a given cognitive impairment than the same lesion atom of the other hemisphere. Hemisphere-specific predictions showed relevant differences for a total of 4 out of 10 lesion atoms considering all four target scores.

**Figure 5:**
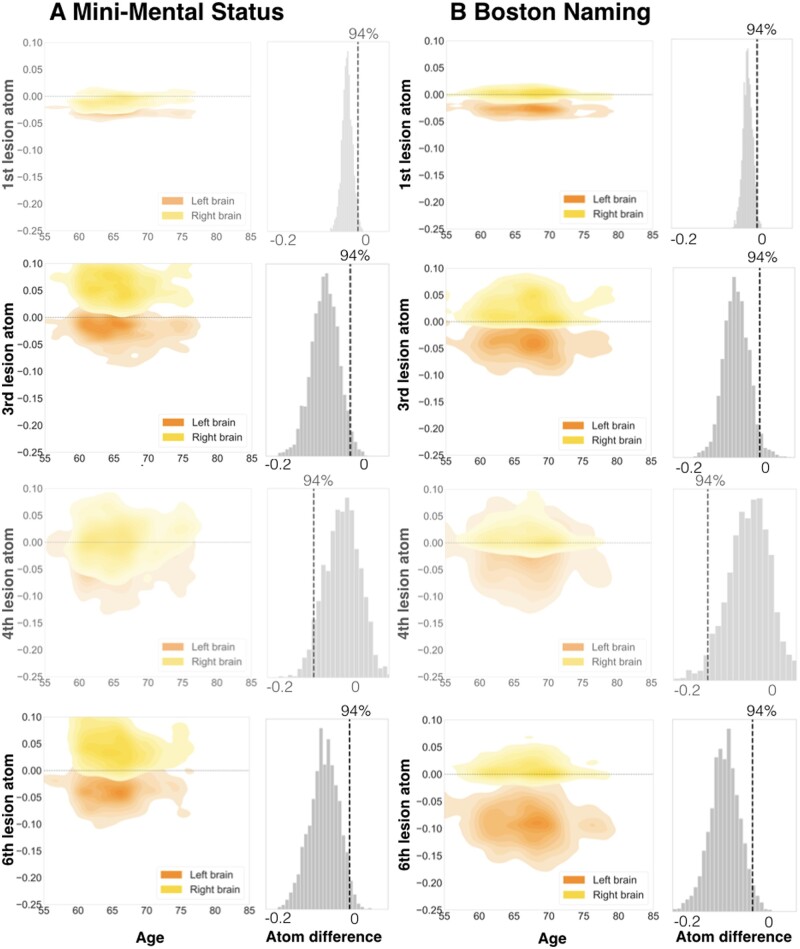
**Specific lesion atoms show strong lateralization for the Mini-Mental State Examination (**A**) and Boston Naming Test (**B**).** Among all candidate lesion atoms, four distributed lesion patterns exerted pronounced hemispheric differences in predictive relevance to forecast future cognitive performance. The Boston Naming (BN) and Seoul Verbal Learning Tests (SVL, c.f., Fig. 6) were characterized by most lateralization effects of three lesion atoms. Two lesion atoms were substantially lateralized for the Mini-Mental State Examination (MMSE). Generally, we derived lateralization effects for lesion atoms by subtracting its marginal posterior distribution of one hemisphere from the marginal posterior distribution of the other hemisphere. We assumed a substantial lateralization when the distribution of the difference did not overlap with zero. In MMSE, hemispheric differences arose from right-hemispheric positive and left-hemispheric negative predictive relevances (positive: predictive of preserved function, negative: predictive of lost function). Left lateralization for BN originated from the difference between negative left-hemispheric and neutral right-hemispheric predictive relevances (exemption: lesion atom 3 was lateralized due to negative left- and neutral right-hemispheric relevances). Left columns: Plots visualize the joint density for combinations of parameter weights for age and each of the four lesion atoms. Age is plotted on the *x*-axis, weights of lesion atoms on the *y*-axis. Left-hemispheric lesion atom weights are shown in orange, right-hemispheric ones are shown in yellow. Right columns: Differences between left- and right-hemispheric lesion atom-wise predictive relevances. Figures are shown slightly transparent, in case of a non-defensible difference and thus absent lateralization (zero inside of 94% highest-posterior density interval).

**Figure 6.**
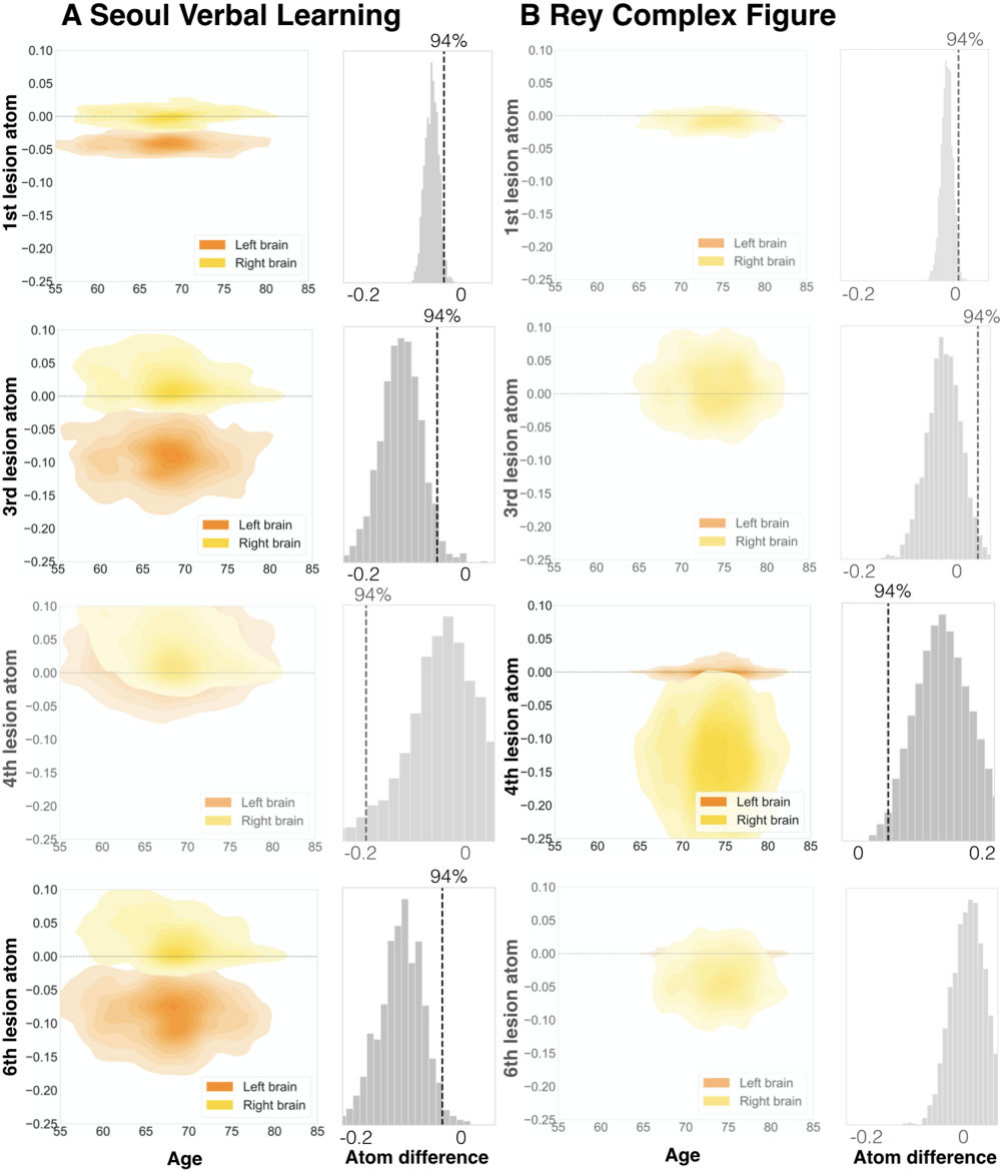
**Specific lesion atoms show strong lateralization for the Seoul Verbal Learning (**A**) and Rey Complex Figure Test (**B**).** The Seoul Verbal Learning Tests (SVL) featured lateralization effects for three lesion atoms, as did the Boston Naming Test. Only one lesion atom was lateralized for the Rey Complex Figure Test (RC). Left lateralization for SVL arose due to the difference between negative left-hemispheric and neutral right-hemispheric predictive relevances. Conversely, right lateralization for RC occurred due to negative right-hemispheric and neutral left-hemispheric predictive relevances. Left columns: Plots visualize the joint density for combinations of parameter weights for age and each of the four lesion atoms. Age is plotted on the *x*-axis, weights of lesion atoms on the *y*-axis. Left-hemispheric lesion atom weights are shown in orange, right-hemispheric ones are shown in yellow. Right columns: Differences between left- and right-hemispheric lesion atom-wise predictive relevances. Figures are shown slightly transparent, in case of a non-defensible difference and thus absent lateralization (zero inside of 94% highest-posterior density interval).

The first lesion atom was characterized by a pronounced left-lateralization of predictive relevance for BN and SVL (BN: hemispheric difference in the posterior mean = −0.029, HPDI = −0.0529–0.00162; SVL: difference posterior mean = −0.0421, HPDI = −0.0711–0.0132). While the posterior distributions for the right-hemispheric first lesion atom did not meaningfully differ from zero, the left-hemispheric lesion atom distributions featured a pronounced deviation in the negative direction: Signalling that only lesions in the left lesion atom 1 regions were predictive of lost function in the BN and SVL (Figs 5 and 6, upper rows; Supplementary Fig. 2, upper left panel). The topography of the first lesion atom covered especially lesions of the globus pallidum and amygdala. Lesion atom 3 combined insular and opercular cortex, Heschl’s gyrus and the putamen and comprised lateralization effects for the MMSE, as well as BN and SVL (MMSE: difference posterior mean = −0.0822, HPDI = −0.155–0.00853; BN: difference posterior mean = −0.0681, HPDI = −0.138–0.001; SVL: difference posterior mean = −0.116, HPDI = −0.211–0.0334). In case of SVL, the right-hemispheric posterior distribution once again did not diverge from zero and the left-hemispheric posterior distribution showed a marked shift into the negative direction. For the MMSE and BN, the difference emerged due to lesions in the right hemisphere being slightly more predictive of preserved function and lesions in the left hemisphere being more predictive of lost function. For RC, we witnessed lateralization effects to the right within lesion atom 4 that highlighted lesions affecting supramarginal, angular and Heschl’s gyrus as well as the superior parietal lobule and lateral occipital cortex (difference posterior mean = 0.137, HPDI = 0.006–0.261). Lastly, MMSE, BN and SVL led to marked left-lateralized predictive relevance in lesion atom 6 that comprised superior, middle and inferior temporal gyri, as well as lateral occipital cortex, planum temporale and amygdala (MMSE: difference posterior mean = −0.0837, HPDI = −0.165 to 0.0107; BN: difference posterior mean = −0.103, HPDI = −0.19–0.0241; SVL: difference posterior mean = −0.117, HPDI = −0.213-0.0214).

### Region relevance for clinical outcome

**Figure 7.**
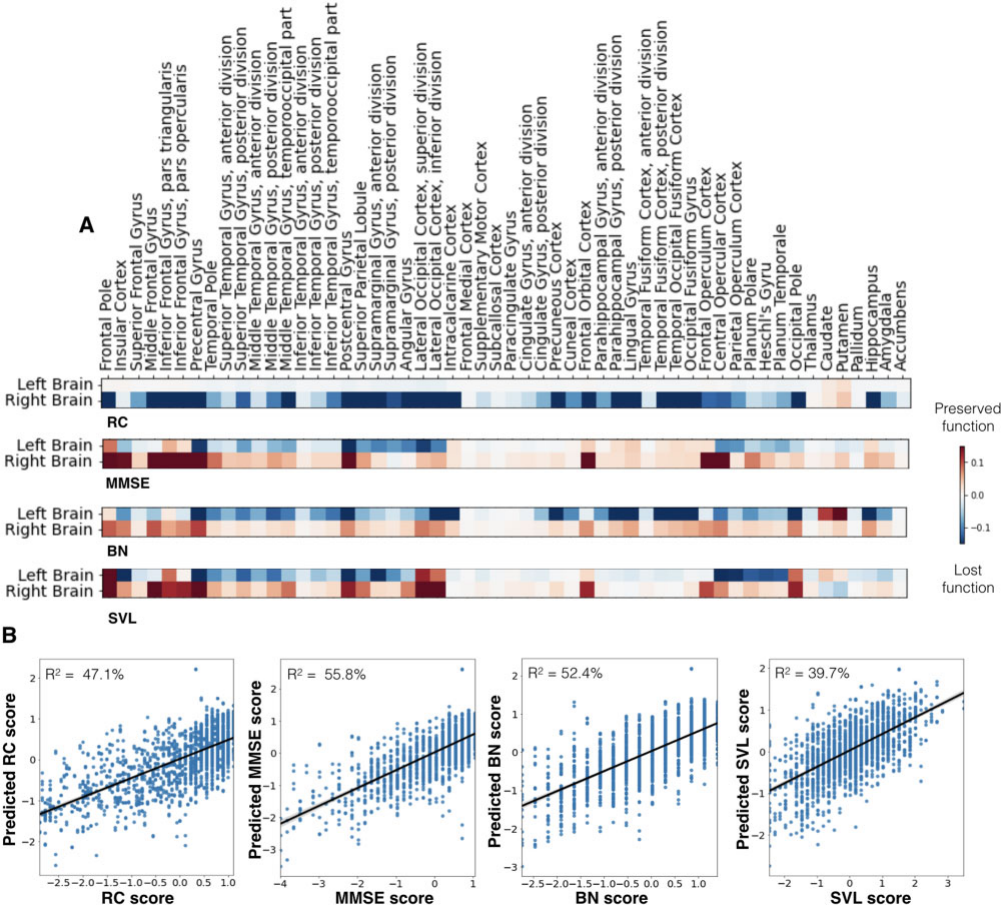
**Cognitive impairments caused by stroke were predicted by atlas regions with unique lateralization effects.** (**A**) Heatmaps indicate the associations of each anatomical region (Harvard-Oxford atlas)^28^ with lost (*blue colour*) or preserved (*red colour*) clinical outcomes in patients. The Rey Complex Figure Test (RC) exclusively featured right-hemispheric predictive relevances. Lesions of right pre- and post-central gyrus, lateral occipital cortex, the occipital pole and hippocampus had the greatest predictive relevance of lost function. The Mini-Mental State Examination (MMSE) stuck out amongst all four scores as it featured predictive relevances that were equally prominent in the left and right hemisphere. However, these relevances were mainly indicative of preserved function (*red*) in the right hemisphere and of lost function on the left hemisphere (*blue*). The Boston Naming (BN) and Seoul Verbal Learning Test (SVL) were both characterized by more pronounced left-hemispheric predictive relevances. These two tests differed in the exact region-wise distribution of the predictive relevances: lesions predictive of poorer naming function primarily extended from the mediotemporal hippocampal to further occipital areas, while verbal learning impairments were better predicted by lesions affecting opercular and insular cortices, as well as planum polare and temporale. (**B**) Prediction accuracy (*R*^2^ estimated based on posterior predictive checks) for the four cognitive scores along with scatterplots of actual (*x*-axis) and predicted (*y*-axis) cognitive performances. The MMSE model achieved the highest *R*^2^ prediction performance (55.8%). In view of 52.4% and 47.1% explained variance, the BN and RC model reached slightly lower scores. The explained variance of the SVL model totalled 39.7%.

Coherent with our hemisphere-specific findings, we uncovered comparable predictive relevances at the brain-region level of the same estimated Bayesian model. Regarding MMSE, lesioned regions on the left were generally more predictive of lost function (negative parameter weights), while lesioned regions on the right were more predictive of preserved global cognitive functions (positive parameter weights). Explained variance, as determined via predictive posterior checks, for the MMSE model was *R*^2^ = 55.8% (Fig. 7B, middle left panel).

Among the 108 total regions for both hemispheres, top regions informative for the prediction of lost MMSE function were the supramarginal and angular gyrus, the pre- and post-central gyrus as well as the lateral occipital and opercular cortices. To a lesser degree, the insular cortex and superior and middle temporal gyri contributed to the prediction of lost function as well (Fig. 7A and Supplementary Fig. 3, second rows). Crucially, all of these areas were located in the left hemisphere. Lesions located in frontal areas of both hemispheres as well as right-hemispheric pre- and post-central and latero-occipital lesions were predictive of comparably more preserved function.

Regarding BN, predictive lesion patterns were predominantly isolated in the left hemisphere. The full model explained *R*^2^ = 52.4% of the variance in the BN outcome (Fig. 7B, middle right panel). The extent of naming impairment was particularly predicted by lesions extending from the mediotemporal hippocampal to further occipital areas. Moreover, pre- and post-central gyri, as well as extended parts of the temporal gyrus, especially superior and middle temporal gyri, demonstrated predictive relevance of lost function (Fig 7A and Supplementary Fig. 3, third rows).

Regarding SVL, predictive lesion patterns of lost function once again related to left-hemispheric lesions, while frontal lesions as well as those of the right hemisphere were associated to a more preserved function. Posterior predictive checks indicated an explained variance of *R*^2^ = 39.7% (Fig. 7B, right panel). Left-hemispheric opercular and insular cortices, as well as planum polare and temporale and to a lesser degree left hippocampal areas predicted deteriorated SVL function (Fig. 7A and Supplementary Fig. 3, bottom rows).

With respect to RC, a distinctly different predictive pattern compared to the previous three scores was observable: while left-hemispheric lesions did not contribute to the prediction in neither the positive, nor the negative direction, right-hemispheric lesions were linked to more deteriorated performance, the explained variance was at *R*^2^ = 47.1% (Fig. 7B, left panel). The highest weights predictive of lost function were assigned to the right middle frontal gyrus, pre- and post-central gyri, lateral occipital cortex, the occipital pole and hippocampus (Fig. 7A and Supplementary Fig. 3, top rows).

Results remained essentially the same when reducing the entire sample to only those patients above the age of 65 years (Supplementary Fig. 4). When conjointly analysing BN and SVL performances, the left hemisphere once again emerged as more relevant hemisphere and region-wise results resembled the ones for the BN test (Supplementary Fig. 5).

## Discussion

The majority of unique constellations of brain tissue damage show some extent of specific cognitive impairments.^2^ We here demonstrate the value of Bayesian hierarchical frameworks to explicitly model several biologically meaningful levels of stroke pathology to explain interindividual differences in cognitive outcomes. We automatically distilled plausible cortical/subcortical lesion patterns from a large multisite sample of 1080 stroke patients. These lesion configurations, naturally recurring motifs across patients, faithfully revealed coherent lesion atoms for cortical and subcortical middle cerebral artery infarction, as well as lesion patterns recapitulating the supply territories of the anterior and posterior cerebral artery.

Our generative modelling tactic enabled the conjoint appreciation of predictive lesion features at the level of brain hemispheres, spatially distributed lesion atoms and individual brain regions. At the hemisphere level of our hierarchical model, we confirmed existing evidence for substantial lateralization effects in the left brain for naming and verbal learning impairments and in the right brain for visuospatial functions after stroke. For more global measures of cognitive performance, quantified by the MMSE, both hemispheres turned out to be equally informative about the patients’ clinical profiles. Importantly, the left hemisphere was predictive of comparatively more deteriorated performance, while the right hemisphere predicted less deterioration. At the level of lesion patterns distributed across regions, we found lateralization effects on the left for patterns capturing damage in the inferior frontal gyrus, insular and opercular cortex, the temporal lobe as well as the basal ganglia for MMSE, BN and SVL. Lateralization to the right was found for a lesion pattern comprising angular gyrus, superior parietal lobule and lateral occipital cortex in case of RC. At the level of single brain regions, naming and memory impairment predictions were especially supported by left-hemispheric and visuospatial impairment predictions by right-hemispheric PCA-supplied brain regions, which involved damage to either the left or right hippocampus, respectively.

Our analytical approach departs from classical lesion-symptom mapping efforts in several important ways. Previous stroke imaging research oftentimes pursued approaches that explore the effect of one brain location at a time.^9^^,^^38^ Analysing brain locations one after another however renders this classical approach less biologically meaningful by omitting lesion interactions between brain locations, which can be spatially distant. Fitting one model per voxel also renders this voxel-wise lesion symptom mapping methodology unsuitable for deriving overall estimates of clinical outcome predictions. Patient-specific predictions are more readily addressed by a single quantitative model encompassing the whole brain-imaging-derived information. By fitting one model to the grey matter of the entire brain, our proof-of-principle approach simultaneously appreciates effects in (and inter-relations between) local regions, their spatially distributed lesion constellations and hemispheric idiosyncrasies in how they relate to clinical outcomes. Together, our approach bears some resemblance with recently introduced multivariate lesion-symptom mapping methods that also take into account entire lesion topographies instead of single voxels.^12^^,^^15^^,^^16^ While such machine learning-based approaches are typically committed to the optimization of prediction performances in a frequentist, point-estimate-focused fashion, our main aim here was to model lateralization effects and their links to cognitive performances in a probabilistic multi-level framework.

From a broader perspective, lateralization is a particularly well-established clinical feature for two frequent cognitive impairments after stroke. Each of these conditions is seen in at least 40% of common acute stroke patients: spatial neglect^13^ and aphasia.^39^ In numerous cases, previous stroke studies cannot truly evaluate such effects due to a priori selection of anatomical regions of interest in either the left or right hemisphere.^8^ Furthermore, many lesion studies threshold their segmented lesion maps to focus on brain regions that are affected in more than 5–10% of patients at hand.^12^^,^^40^ This common practice also often amounts to confining analysis and interpretation to regions from one half of the brain.

To complement these previous research approaches, we aimed to avoid restricting analyses to one hemisphere or measuring voxel-wise lesion load. Instead, we established a principled analysis framework to capture hemispheric predictive relevance as a function of the cognitive outcome at hand in a dedicated probabilistic parameter estimate. In view of this single estimate for the total impact of one brain side, this quantification of hemispheric relevance enabled us to put lateralization-focused interpretations on a firmer basis. In so doing, we witnessed strong left-lateralization for BN and SVL, and right-lateralization for RC. Both the BN and SVL target the evaluation of memory functions, yet also heavily rely on language functions.^20^^,^^22^ Thus, the observed left-lateralization may well reflect these language functions’ main location in our subjects’ left hemispheres. Conversely, we did not find any evidence of hemispheric predominance, rather equally distributed predictive relevances across both hemispheres, for the MMSE. This balanced contribution of both hemispheres in MMSE resulted from left-hemispheric lesions being predictive of more deteriorated test performance, while right-hemispheric lesions were predictive of more preserved function. Therefore, the relation between the left hemisphere and test performance resembled the one extracted for BN and SVL. Given that we focused on predictability and not causality^^70^^, our finding does not imply an improved MMSE test performance in case of right-hemispheric lesions, yet merely a consistently less deteriorated performance compared to patients with left-hemispheric lesions. Also, the prediction of more preserved MMSE performance for lesions in the right hemisphere might be erroneously caused by the MMSE’s insensitivity to cognitive deficits after right-hemispheric lesions, as frequently reported in the literature.^41–43^ In particular, the MMSE was found to be incapable of capturing impairments in abstract reasoning, executive functioning, and visual perception.^44^ Altogether, it might thus not represent a reliable screening test for cognitive impairments in stroke patients.

While we found left-lateralization for cortical regions classically known to subserve lateralized language function, we also discovered indicators of left hemisphere-specific effects in subcortical regions. More precisely, these effects of lateralization highlighted especially the pallidum and putamen.

Aphasia is traditionally viewed as a deficit of the cortical mantle. Nonetheless, speculations about an involvement of the basal ganglia go back to the late 19th century.^5^^,^^45^^,^^46^ The exact role of non-thalamic subcortical areas in language functions remains incompletely understood, despite various lesion-symptom and functional neuroimaging studies in small cohorts. Characterizations of subcortical aphasias are highly heterogeneous and structure-function correspondences were repeatedly found to be weak.^47–49^ Our lesion findings are coherent with the interpretation of an implication of left putamen and left globus pallus in healthy language and verbal memory function.

Several investigators have described declines in verbal learning and memory functions specifically after left-sided pallidotomy: For instance, Lang and colleagues^50^ noted word finding impairments in 69% of patients after left-sided pallidal lesions, yet only in 25% of patients with such lesions on the right. Similarly, further studies suggested a higher verbal memory impairment after left- than after right-sided pallidotomy.^51^^,^^52^ However, the majority of these studies relies on less than 70 total patients. Moreover, analyses were previously restricted to the evaluation of anatomically very circumscribed lesions. In our approach, we extracted lesion atom-wise probabilistic hemispheric effects in 1080 patients while accounting for all other extracted lesion atoms covering regions across the entire brain. By these means, we can add convincing evidence to previously small and locally restricted studies.

Leveraging our novel, hemisphere-aware modelling approach, we could also reliably confirm and substantially expand previous reports on certain individual brain regions: For MMSE, BN and SVL, we found predictive contributions to function loss for the superior, middle and inferior temporal gyrus, as well as insular and angular regions on the left side of the brain. Consistent with our present findings, these regions were also outlined in previous lesion-symptom studies of global cognitive and naming functions.^10^^,^^53–55^ Further, limbic system regions, such as the hippocampus and amygdala as observed here, are well known to be implicated in memory functions in the intact human brain.^56^ Previous lesion-symptom mapping analyses of visuospatial functioning pointed to right-hemispheric frontal lobe, superior temporal lobe and supramarginal gyrus regions,^57^ as well as lingual, fusiform and inferior parietal cortices,^58^ which we also detected in our analyses.

Most previous work presuming lateralization has focused on language or attentional impairments related to neglect. Due to their anatomical localization, these impairments closely relate to middle cerebral artery strokes. That is, strokes in the most commonly affected vascular territory.^59^ Adding to these previous efforts, we here also identified predictive contributions of lesions in the less commonly affected vascular supply territory of the PCA. The here uncovered brain regions exerted negative effects on naming and memory functions if affected in the left hemisphere and on visuospatial abilities if affected in the right hemisphere. All of these posterior regions were collectively represented by one of our automatically extracted lesion atoms. The hippocampus emerged among the implicated regions in this specific lesion atom, which matched its frequent blood supply by the PCA.^60^

A few earlier observations in stroke patients exist that have hinted at pronounced cognitive impairments after only left-sided stroke lesions due to PCA occlusions.^61–63^ More specifically, in neuropsychological testing, patients with PCA stroke performed particularly poorly in naming tasks and verbal memory tests.^61–63^ A study that contrasted 20 left- and right-hemispheric PCA-stroke patients reported no differences in MMSE performance. However, the investigators noted significantly lower performances of patients with left- versus right-hemispheric stroke in two verbal learning tests.^63^ Importantly, all patients considered in this study had lesions exclusively in the PCA territory and all involved hippocampal regions. Further studies described the involvement of PCA territory regions for verbal memory,^16^ and visuospatial functions,^58^ however without a clear-cut lateralization predilection.

Our study strengthens these previous findings by carefully quantifying the extent of cognitive impairments as a general consequence of brain infarcts extending to the left or right PCA territory in our sample of 1080 patients. Our results are coherent with the conclusion that lesions in left PCA-supplied regions have a particularly deleterious effect on naming and memory functions, and on visuospatial functioning if affected in the right hemisphere. In fact, these impairments might primarily relate to hippocampal lesions, as captured in our lesion atom with PCA coverage. Only recently, it was asserted that interindividual differences in several cognitive domains, such as verbal learning and global cognition, could arise from natural variability in vascular supply anatomy of the hippocampus.^64^ These authors argued that a mixed supply, combining vessels from anterior as well as PCA, was more protective of cognitive impairment than a solely PCA-dependent supply. This study relied on data from healthy older adults as well as patients with cerebral small vessel disease, which promises future translation to stroke patients and their cognitive recovery.

### Limitations and future directions

Stroke lesions are known to often affect the white matter of the brain.^36^^,^^59^^,^^65^^,^^66^ Previous stroke research has shown that including information on white matter damage can enhance outcome predictions.^16^^,^^17^^,^^67^^,^^68^ We here considered white matter lesions only indirectly by integrating the total lesion volume, i.e., of grey and white matter, as a covariate in our modelling framework. Our main interest in this study was to infer lateralization effects of direct lesions to cortical and subcortical grey matter regions. As such, we, in return, accepted a potentially suboptimal prediction performance—a trade-off, which has been referred to as the prediction-inference dilemma.^69–71^ Future studies could however choose to add the explicit modelling of white matter effects by quantifying white matter damage within white matter tracts, as was recently done in a lesion-symptom mapping study of acute stroke severity.^72^

Similarly, while beyond the scope of the current work, a promising approach for future research would be to phenotype a large stroke sample, as investigated here, in even greater detail in order to measure fingerprints of small vessel disease, such as white matter hyperintensities, lacunar infarctions, and cerebral atrophy. This avenue will be key given these factors’ intricate associations with vascular cognitive impairment.^73–75^ For example, the presence of multiple silent lacunar infarcts, which was accompanied by a higher white matter hyperintensity load, could be linked to more prominent neuropsychological abnormalities.^75^ Also, it was shown that subcortical ischaemia could lead to secondary neurodegeneration of cortical regions because focal thinning was observed for cortical regions connected to subcortical lesions.^74^ Indeed, patients with lacunar infarctions and cognitive impairment featured more pronounced atrophy in several bilateral cortical regions, such as middle temporal gyrus, frontal and posterior occipitoparietal regions, when compared to patients with lacunar infarctions but without cognitive impairment.^73^

With respect to our data analysis workflow, future research may explore further variations. For example, the discovery of lesion patterns could employ non-linear dimensionality reduction techniques, such as t-distributed stochastic neighbor embedding (t-SNE)^76^ or Uniform Manifold Approximation and Projection (UMAP),^77^ that were recently shown to be fruitful for deriving informative lesion representations.^78^ Our focus here was on neurobiological inference of hemisphere- and region-specific lateralization effects. Yet, more prediction-focused studies^69–71^ could also replace our NMF step with a principal component analysis, which has been shown to be a constructive pre-processing approach in prediction-focused scenarios.^15^^,^^16^

Lastly, such more prediction-focused studies could also further investigate differences in explained variances. We here observed *R*^2^-based explained variances from a minimum of 39.7% (SVL) to 55.8% (MMSE). Conceivable, these differences may originate from a multitude of different sources, such as differences in the variability of outcome scores related to the lesion anatomy and more or less extractable brain-behaviour associations. Altogether, our current levels of explained variance may also motivate the inclusion of even more biomarkers of stroke impairment and recovery in addition to the ones considered here.^79^

## Conclusion

We have introduced a probabilistic analytical framework that pools information across the entire brain and can therefore simultaneously integrate lesion facets on varying types and forms. Our results highlight strong lateralization effects to the left for naming and memory functions, and the right for visuospatial functions. These asymmetry effects were related to cortical and also subcortical regions, such as the pallidum and hippocampus, supplied by the middle and posterior cerebral artery vascular territories. Future studies, that employ the probabilistic multi-level approach as showcased here, are warranted to further elucidate hemispheric inter-relations for complex cognitive functions, such as spatial and verbal memory, and eventually finesse the probabilistic prediction of cognitive impairment at the single patient level.

## Supplementary Material

fcab110_Supplementary_Data

## Abbreviations

BN =: Korean short version of the Boston Naming
DWI =: diffusion-weighted imaging
HPDI =: highest probability density interval
IQCODE =: Informant Questionnaire on Cognitive Decline in the Elderly
MMSE =: Mini-Mental State Examination
NMF =: non-negative matrix factorization
PCA =: posterior cerebral artery
RC =: Rey Complex Figure Test
SVL =: Seoul Verbal Learning Test
