# Correction: Generative lesion pattern decomposition of cognitive impairment after stroke

**DOI:** 10.1093/braincomms/fcad154

**Published:** 2023-05-16

This is a correction notice for: Anna K Bonkhoff, Jae-Sung Lim, Hee-Joon Bae, Nick A Weaver, Hugo J Kuijf, J Matthijs Biesbroek, Natalia S Rost, Danilo Bzdok, Generative lesion pattern decomposition of cognitive impairment after stroke, *Brain Communications*, Volume 3, Issue 2, 2021, fcab110, https://doi.org/10.1093/braincomms/fcab110

In the originally published version of this manuscript, there was an error in a detail for the history of the article published online and in the PDF. In the PDF at the foot of p.1, this detail should read: “Received 28 December 2020” instead of: “Received 28 December 2021”. Online, the detail and order of the history dates should read:

“Received: 28 December 2020

Revision received: 21 February 2021

Accepted: 20 May 2021

Published: 22 May 2021”

Instead of:

“Revision received: 21 February 2021

Accepted: 20 May 2021

Published: 22 May 2021

Received: 28 December 2021”.

These errors are emended only in this correction notice to preserve the published version of record.

