# Supplementary material for: Generative lesion pattern decomposition of cognitive impairment after stroke
Source: Brain Commun. 2021 May 22;3(2):fcab110. doi: 10.1093/braincomms/fcab110 (PMC8233115; doi:10.1093/braincomms/fcab110)
Supplement: fcab110_Supplementary_Data [file fcab110_supplementary_data.docx]

**Supplementary material**

**Neuroimaging data**

**Seoul National University Bundang Hospital**

| **FLAIR** | repetition time: 11,000 ms; echo time: 125 ms; inversion time: 2,800 ms; slice thickness_ 5 mm; intersection gap: 1 mm; matrix: 512 x 512; flip angle 90 degree |
| --- | --- |
| **DWI** | EPI-spin echo sequence; repetition time: 5,000 ms; echo time: 50 ms; diffusion b-value: 1,000; slice thickness: 5 mm; intersection gap: 1 mm; matrix: 256 x 256; flip angle 90 degree |

**Hallym University Sacred Heart Hospital**

| **FLAIR** | repetition time: 11,000 ms; echo time: 125 ms; inversion time: 2,800 ms; slice thickness: 5 mm; matrix: 512 x 512; flip angle 90 degree |
| --- | --- |
| **DWI** | repetition time: 3,000 ms; echo time: 56 ms; diffusion b-value: 1,000; slice thickness: 5 mm; matrix: 256 x 256; flip angle 90 degree |


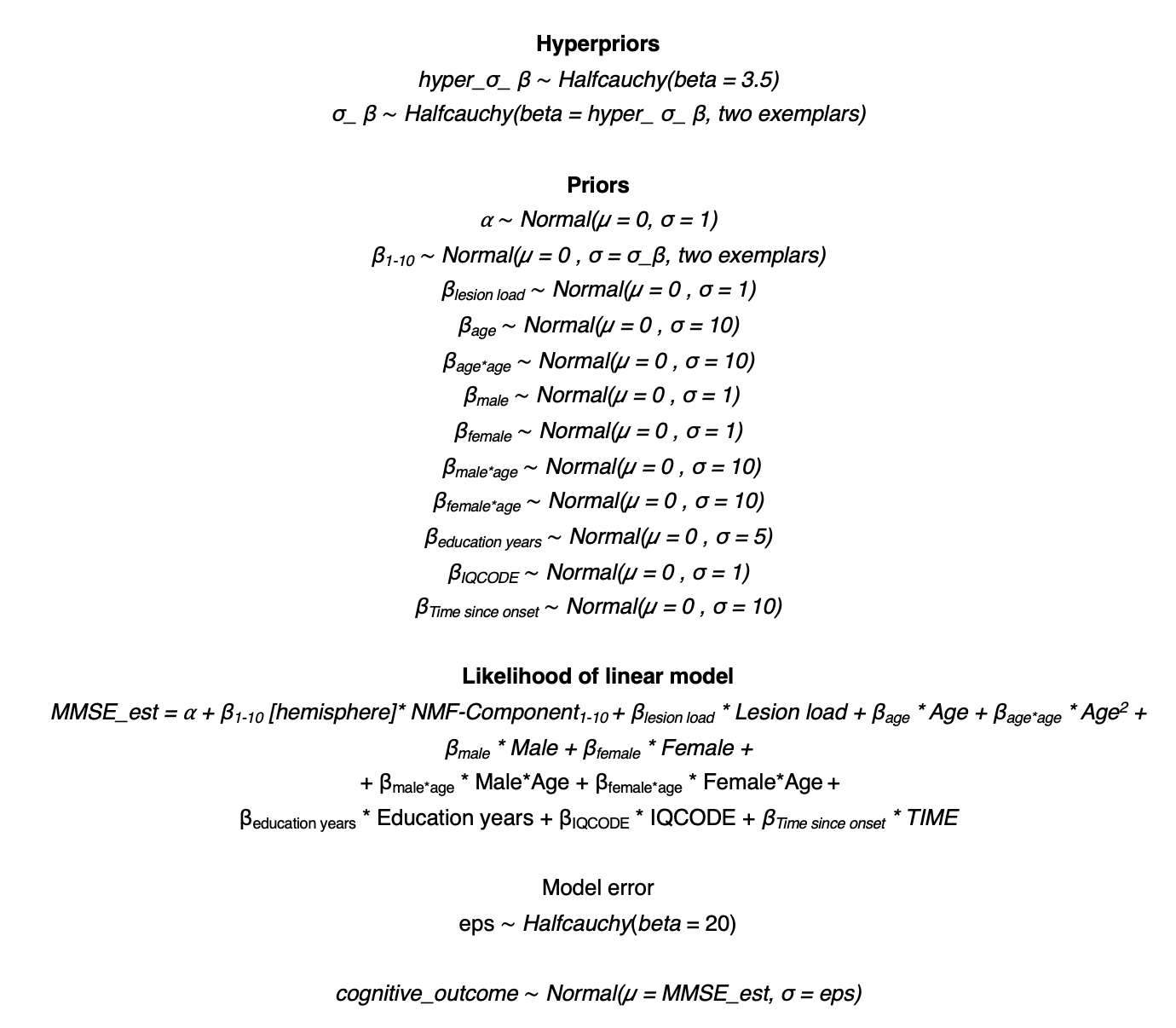


**Supplementary figure 1.** Full Bayesian model specification, exemplarily for MMSE.


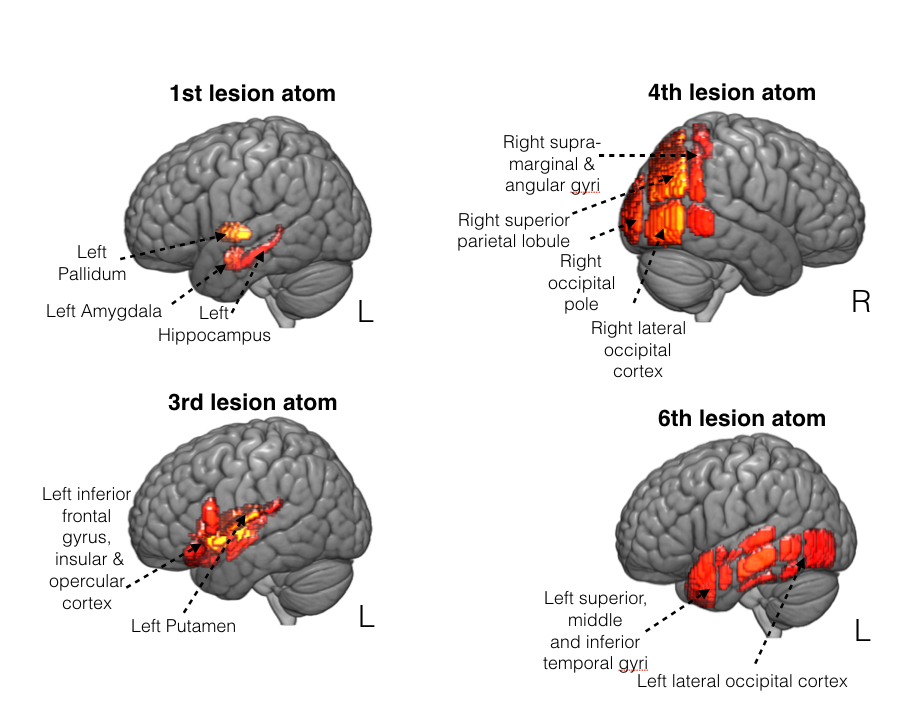


**Supplementary Figure 2. Four lateralized lesion atoms.** The four lateralized lesion atoms predominantly represented subcortical structures (putamen, globus pallidus, amygdala, hippocampus) as well as insular and temporal lobe regions on the left and supramarginal, angular and Heschl’s gyrus as well as the superior parietal lobule, lateral occipital cortex and occipital pole on the right.


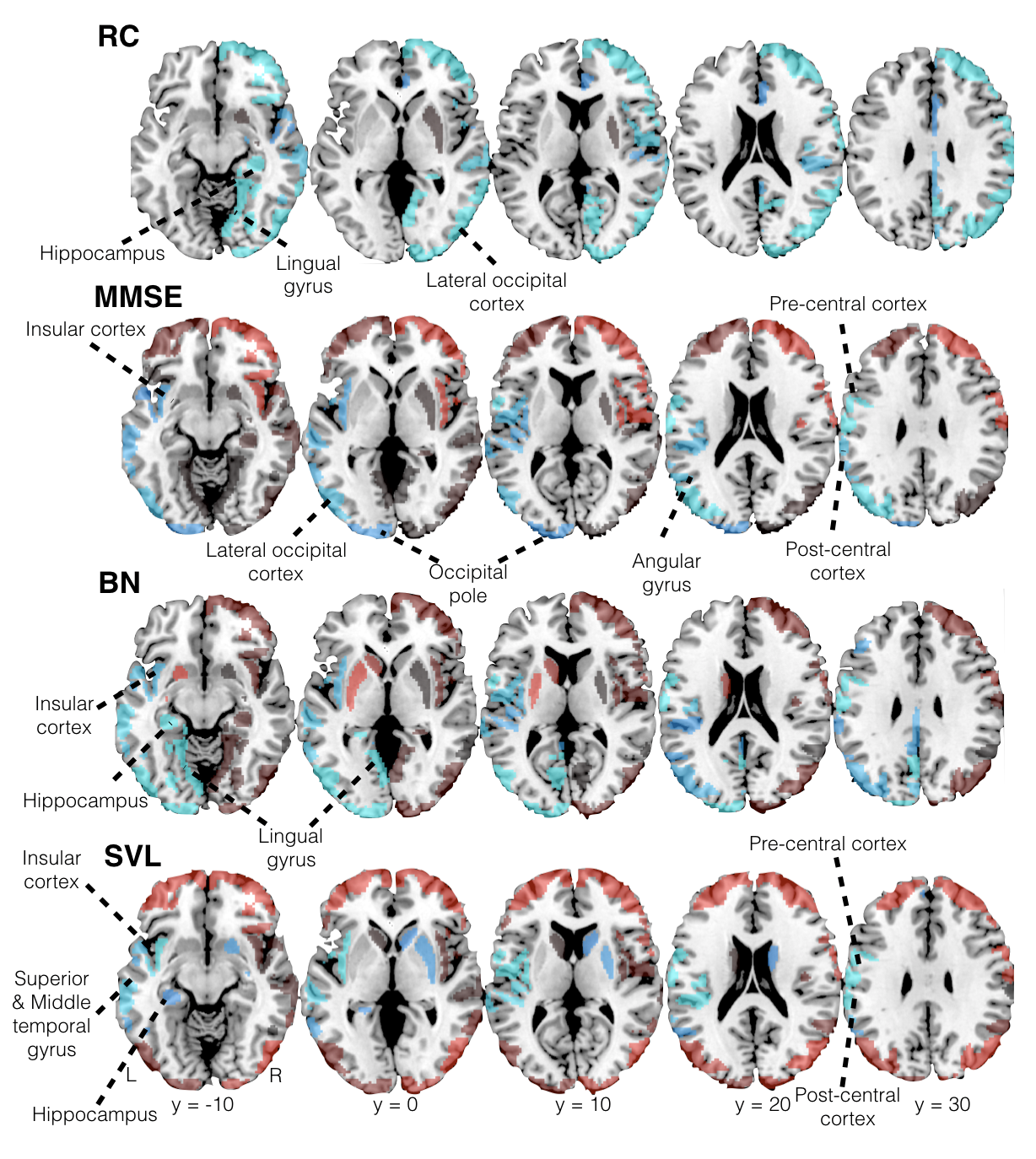


**Supplementary Figure 3.** Brain renderings of the brain region-wise predictive relevance, described more in detail under **Figure 6A** in the main manuscript. Blue colors indicate lost, while red colors indicate preserved function in the four investigated tests (RC: Rey Complex Figure Test, MMSE: Mini-Mental State Examination, BN: Boston Naming Test, SVL: Seoul Verbal Learning Test).


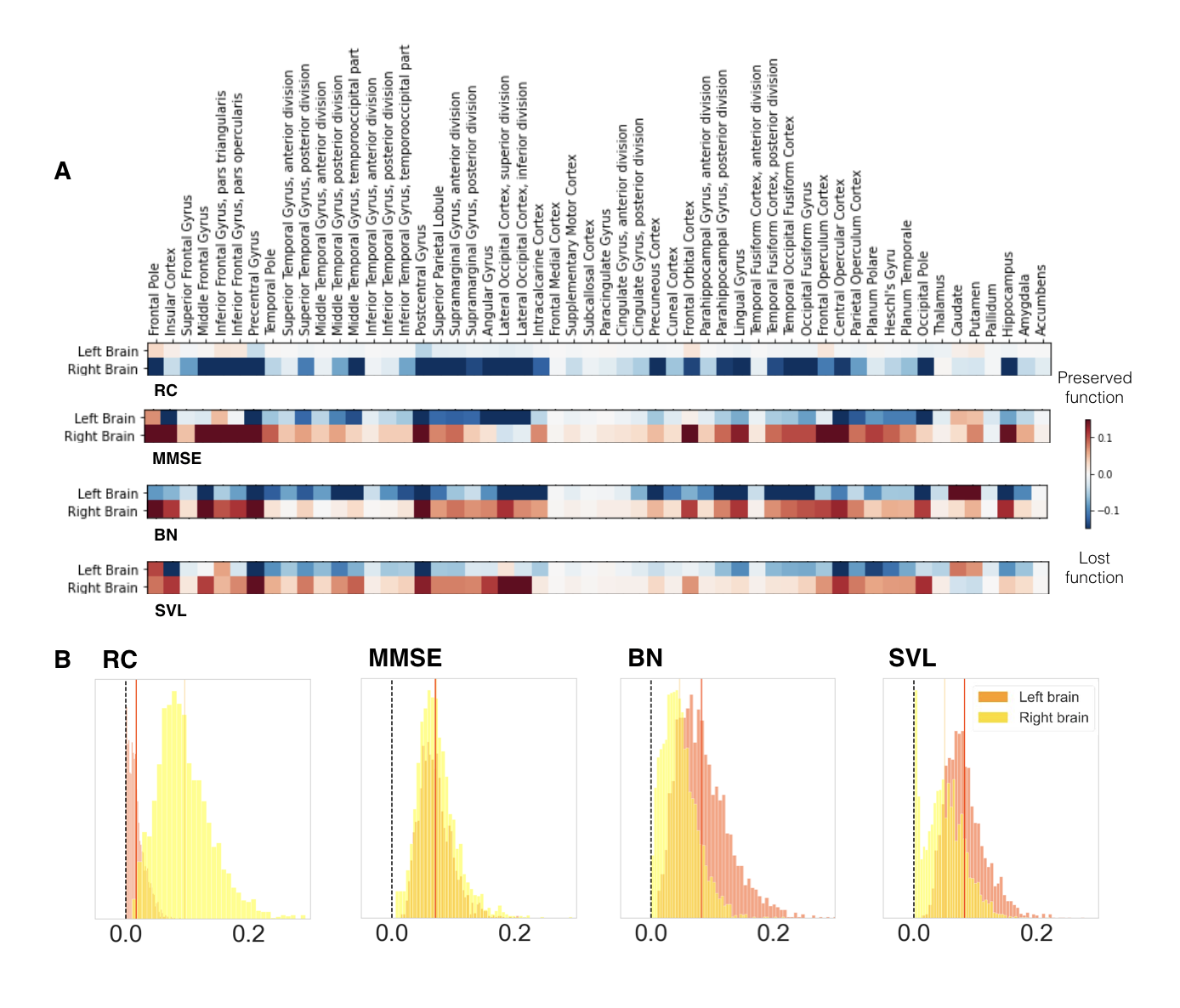


**Supplementary Figure 4.** **Modelling of all four outcome scores for patients above the age of 65 years only** (exemption: above the age of 60 in case of RC). **A. Region-wise relevances. B. Hemisphere-specific standard deviation hyperpriors.**


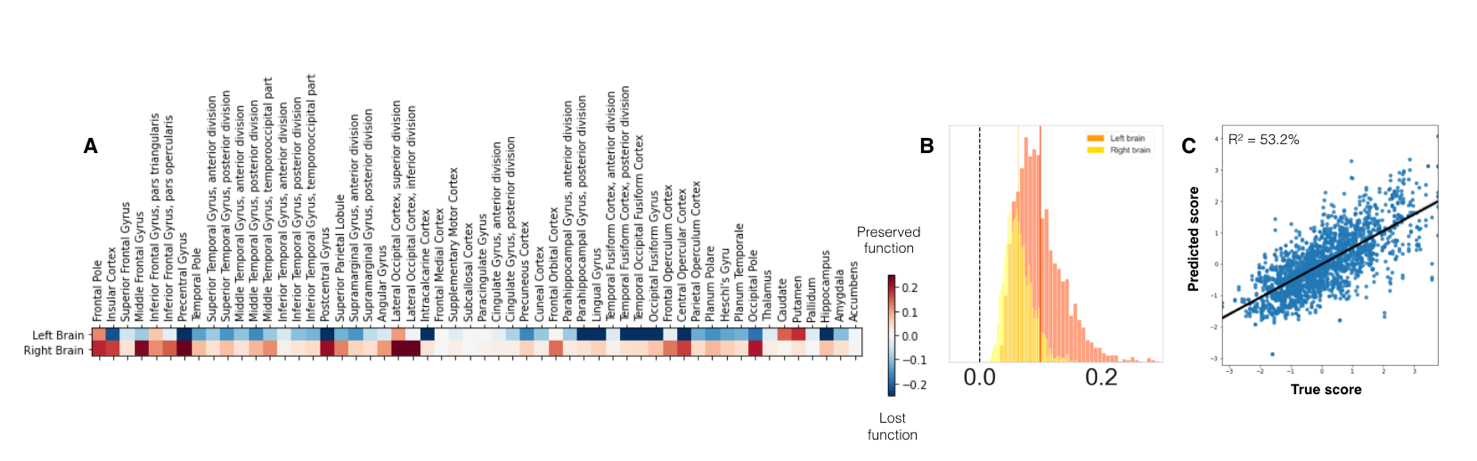


**Supplementary Figure 5. Joint analysis of Boston Naming and Seoul Verbal Learning Test performances. A. Region-wise relevances. B. Hemisphere-specific standard deviation hyperpriors. C. Posterior predictive check derived explained variance.** The correlation of these two highly language-dependent tests was r=0.61. The first component after principal component analysis of both scores explained a variance of 81%.
